# Effect of Iodine Nutrition During Pregnancy and Lactation on Child Cognitive Outcomes: A Review

**DOI:** 10.3390/nu17122016

**Published:** 2025-06-16

**Authors:** Zheng Feei Ma, Louise Brough

**Affiliations:** 1 School of Health and Social Wellbeing, College of Health, Science and Society, University of the West of England, Bristol BS16 1QY, UK; 2 School of Food Technology and Natural Sciences, College of Sciences, Massey University, Palmerston North 4410, New Zealand; l.brough@massey.ac.nz

**Keywords:** iodine, iodine deficiency, excessive iodine, child cognition, pregnancy, lactation

## Abstract

Iodine deficiency remains one of the most serious global public health challenges, recognised as the leading cause of preventable brain damage worldwide. It is widely accepted as the primary aetiological factor underlying iodine deficiency disorders (IDD). Inadequate maternal iodine intake reduces thyroxine synthesis, impairing foetal brain development and leading to long-term deficits in cognitive function across childhood and adulthood. However, emerging evidence also suggests that excessive iodine intake may disrupt thyroid function, particularly in individuals with underlying thyroid autoimmunity, potentially leading to adverse neurodevelopmental outcomes. In this state-of-the-art review, we examine the effects of iodine nutrition during pregnancy and lactation on child cognitive outcomes. We provide an overview of the recent global iodine status, critically appraise the current evidence linking both iodine deficiency and excess to neurodevelopmental outcomes, and offer expert interpretation of the key findings. We further highlight areas of uncertainty, introduce emerging evidence from contemporary studies, and propose directions for future research to inform and optimise public health policies and clinical practice. Our findings highlight a U-shaped association, whereby both insufficient and excessive iodine exposure during pregnancy and lactation may impair optimal brain development in the offspring.

## 1. Introduction

Iodine is a critical trace element essential for the synthesis of the thyroid hormones (triiodothyronine (T3) and thyroxine (T4)), which are necessary for normal foetal brain development [1,2]. Global dietary guidelines, including those from the WHO, recommend a daily iodine intake of 150 μg for adults, increasing to 250 μg during pregnancy to meet the physiological demands of increased maternal thyroid hormone production and the complete foetal dependence on the maternal iodine supply [3]. Despite these established recommendations, pregnant women remain at an increased risk of iodine deficiency, particularly in industrialised countries where iodine-rich foods are increasingly under consumed [2,4]. This gap between physiological requirements and dietary intake underscores the need to critically evaluate the current strategies for ensuring adequate iodine nutrition during pregnancy, particularly in populations previously considered iodine sufficient.

Pregnant women are widely recognised as a population vulnerable to iodine deficiency, with numerous studies reporting average dietary intakes during pregnancy that fall substantially short of the recommendations [5,6,7,8]. This persistent inadequacy is not solely due to dietary insufficiency but may also reflect a broader lack of awareness and understanding about iodine nutrition, including limited knowledge of the key dietary sources and the role of iodine in foetal development. Such knowledge gaps have been shown to correlate with suboptimal urinary iodine concentrations, the primary biomarker of iodine status. Particularly, studies in high-income countries such as the UK, Australia, and Norway have consistently documented poor iodine-related knowledge among pregnant women, despite public health efforts to improve maternal nutrition [9,10,11]. These findings highlight the need for targeted educational interventions alongside nutritional strategies to address this preventable micronutrient deficiency.

Iodine status in lactating women is also a key public health concern, as it directly influences the iodine content of breast milk and, consequently, the iodine intake of breastfed infants. The iodine status among lactating women demonstrates significant geographical variation, with surveys and cohort studies revealing contrasting trends. In high-income countries such as Norway and Australia, evidence indicates that lactating women often exhibit mild to moderate iodine deficiency [12,13]. This is attributed to factors including the reduced use of iodised salt, dietary changes with a lower intake of iodine-rich foods (such as dairy and seafood), and the absence of routine iodine supplementation policies during lactation. In contrast, studies from China have reported an adequate iodine intake among lactating women, largely due to sustained universal salt iodisation policies and higher baseline iodine consumption [14,15,16]. These disparities underscore the importance of region-specific public health strategies, continuous monitoring, and education to ensure iodine sufficiency during lactation and to protect infant neurodevelopment. Therefore, comprehensive monitoring strategies and tailored interventions are essential to ensure iodine sufficiency during lactation.

Iodine deficiency during pregnancy and lactation is defined according to the urinary iodine concentration (UIC), with WHO/UNICEF/ICCIDD (now Iodine Global Network) guidelines specifying that a median UIC of <150 μg/L in pregnant women and <100 μg/L in lactating women indicates an insufficient iodine intake at the population level (Table 1) [3]. UIC is a useful biomarker for recent iodine intake, as more than 90% of ingested iodine is excreted in the urine. However, its inherent variability at the individual level limits its reliability for assessing the long-term iodine status. Dietary assessment methods, such as the iodine-specific food frequency questionnaire (FFQ) and 24 h dietary recall, offer complementary information by capturing habitual intake patterns and the contribution of iodine-rich foods. When combined, urinary iodine measures (including UIC or iodine-to-creatinine ratio (I/Cr)) and dietary data offer a more robust evaluation of the iodine status during pregnancy, enhancing the precision of studies investigating the associations between maternal iodine exposure and child neurodevelopment.

Cognitive development in children encompasses the progression of abilities such as thinking, learning, memory, problem solving, and language skills [17]. This development is most rapid and sensitive during the early years of life, making adequate nutrition, especially sufficient iodine intake, essential during prenatal and early postnatal periods [18]. Suboptimal iodine status during the critical windows of development, particularly during gestation and early childhood, may lead to irreversible impairments in neurocognitive function. These deficits, which stem from the thyroid hormone’s essential role in brain maturation, may manifest as reduced intellectual capacity, diminished academic performance, and lower economic productivity in later life [18].

Therefore, in this review, we will first discuss the importance of iodine during pregnancy and lactation. Next, we will explore and synthesise the current evidence on both maternal iodine deficiency and excess, evaluating their respective associations with child neurodevelopmental outcomes. By critically appraising the methodological strengths and limitations of the existing studies and their findings, we aim to provide a comprehensive understanding of the relationship between maternal iodine status and cognitive development in children. Additionally, we aim to highlight the importance of optimising the maternal iodine nutrition and inform public health strategies aimed at improving maternal and child health.

### Search Methodology

To critically synthesise the current scientific literature on maternal iodine nutrition and child cognitive outcomes, a structured search strategy was employed across PubMed, Scopus, and Web of Science databases. Relevant peer-reviewed articles published up to 2025 were identified using combinations of key terms such as “iodine”, “iodine deficiency”, “iodine nutrition”, “thyroid function”, “pregnancy”, “lactation”, “child”, “children”, “neurodevelopment”, “child cognition”, and “child development”’. Studies were selected based on their contribution of original data relevant to the maternal iodine status and its child health outcomes. Non-empirical publications, including editorials, commentaries, and conference abstracts lacking primary data, were excluded to ensure the scientific robustness and reliability of the evidence base informing this review.

## 2. Physiology of Iodine During Pregnancy and Lactation

The physiology of iodine undergoes significant changes during pregnancy and lactation due to increased maternal and foetal demands [19]. During pregnancy, the maternal iodine requirements rise by approximately 50% owing to several factors [19]. These include the following: increased renal clearance of iodine due to elevated glomerular filtration rate, enhanced maternal thyroid hormone production to support both maternal and foetal needs, and iodine transfer to the developing foetus, particularly during the second and third trimesters when the foetal thyroid begins hormone synthesis [19]. Iodine is essential for the production of T3 and T4, hormones critical for foetal brain development. During the first trimester, the foetus is wholly dependent on maternal T4, as the foetal thyroid gland does not become functionally active until approximately 12 weeks of gestation [20].

During lactation, iodine is actively transported into breast milk via the sodium/iodide symporter (NIS) (i.e., a 13-transmembrane domain glycoprotein that is located on the basolateral membrane of thyroid follicular cells) in the mammary gland [21]. This mechanism ensures that breastfed infants receive sufficient iodine to support their thyroid function and continued neurodevelopment. The recommended daily iodine intake for lactating women is higher than during pregnancy, which is 250 µg/day to reflect the need to maintain an adequate iodine concentration in breast milk (ideally 100–200 µg/L) (Table 2) [22]. These physiological adaptations highlight the necessity for adequate iodine intake during both pregnancy and lactation to prevent maternal and infant iodine deficiency, which can have long-lasting consequences on cognitive outcomes [22].

## 3. Maternal Iodine Deficiency

### 3.1. Prevalence

Iodine deficiency during pregnancy remains a significant public health concern affecting both developed and developing countries [19,26]. In a meta-analysis of 61 observational studies by Patriota et al., the global prevalence of insufficient iodine intake among pregnant women was estimated at 53% (95% CI: 47–60%), with substantial heterogeneity across studies (*I*^2^ = 99.8%) [27]. Subgroup analyses by geographic region revealed that the prevalence was notably higher in Europe (69%; 95% CI: 55–82%; *I*^2^ = 99.2%) and Eurasia (69%; 95% CI: 55–84%; *I*^2^ = 98.4%) compared to Asia (46%; 95% CI: 40–53%; *I*^2^ = 99.8%) [27]. When stratified by national iodine status, countries classified as having an insufficient iodine status demonstrated a markedly higher prevalence of an inadequate intake (86%; 95% CI: 78–93%; *I*^2^ = 97.0%) compared to those with sufficient iodine status (51%; 95% CI: 45–57%; *I*^2^ = 99.8%) [27]. These findings underscore the critical role of health system interventions and public health monitoring in sustaining efforts to eradicate iodine deficiency, particularly among vulnerable populations such as pregnant women. At the country level, the highest prevalence rates of insufficient iodine intake were reported in Iran (97%), Ukraine (96%), Turkey (90%), and Norway (90%), highlighting regional disparities and the need for targeted iodine supplementation and fortification strategies [27].

Despite the significant global progress in combating iodine deficiency, Europe remains one of the regions where mild iodine deficiency continues to be the most prevalent. Of the approximately 590 million individuals residing in this region, an estimated 350–400 million either lack access to iodised salt or do not consistently use it even when available [28]. Despite the long-standing public health initiatives, more than half of European countries reported national data on iodine intake during pregnancy, and of these, two-thirds documented insufficient iodine consumption among pregnant women [29]. Importantly, iodine supplementation practices during pregnancy remain inconsistent across Europe, and even where supplementation is common, achieving an adequate iodine status as assessed by a median UIC is not guaranteed.

In Denmark, despite the widespread use of iodine-containing supplements following the introduction of salt iodisation, pregnant women who did not take supplements remained at a significantly higher risk of deficiency (a median UIC of 68 μg/L) compared to supplemented women (a median UIC of 109 μg/L) [30]. Similarly, in Belgium, national surveillance revealed a median UIC of 124 μg/L in pregnant women, falling below the WHO-recommended threshold of 150 μg/L for pregnant women (i.e., iodine sufficiency), despite high rates of supplementation [31]. Furthermore, Belgian data indicated that a suboptimal iodine intake during pregnancy was associated with thyroid hyperstimulation, reflected by elevated serum thyroglobulin (Tg) concentrations in the third trimester compared with the first [32]. In the UK, where no national salt iodisation programme exists, a regional survey by Bath et al. similarly highlighted iodine deficiency among pregnant women, with a median UIC of 85 μg/L [33]. These findings collectively underscored the significant shortcomings in the current iodine deficiency prevention strategies across Europe, even in contexts where supplementation and salt iodisation policies have been implemented [34].

Although improvements have been achieved, particularly in the Central and Eastern European regions, through the iodisation of edible salt for both household and industrial use, challenges persist [35]. Over the past two decades, the use of adequately iodised salt has increased, with approximately 55% of households and industries now using iodised products. Nevertheless, recent dietary trends including declines in the consumption of iodine-rich foods such as milk, dairy products, and seafood have contributed to a resurgence of iodine insufficiency in parts of Europe [36].

In Asian countries, such as Iran and Pakistan, geographic and environmental factors also significantly influence the iodine status. Large mountainous territories present logistical barriers to accessing iodine-rich foods, while inland locations far from marine sources limit the natural iodine availability. For example, a study by Rostami, Beiranvand, and Nourooz-Zadeh reported that 98% of Iranian pregnant women residing in mountainous areas exhibited an insufficient iodine intake during the first trimester, compared to 84% among those living in lowland plains [37]. These findings emphasise the persistent geographical disparities in iodine nutrition and highlight the need for region-specific public health interventions, including tailored fortification strategies and improved food distribution systems.

There remains a significant paucity of data regarding the iodine nutritional status of pregnant women in Africa, despite evidence indicating that approximately 90% of African countries are at risk of iodine deficiency. This vulnerability is largely attributed to the poor soil iodine content and the widespread consumption of dietary goitrogens, which inhibit the iodine uptake and utilisation [38]. Among African nations, Ethiopia, Morocco, and Niger have reported the highest prevalence rates of inadequate iodine intake, conditions closely linked to weak legislative frameworks, insufficient monitoring systems, and broader challenges such as conflict and political instability [39]. These findings suggest that the observed geographic variation in the prevalence of iodine deficiency is influenced by a combination of environmental factors, dietary practices, and the effectiveness of salt iodisation policies. Strengthening national iodine programmes, ensuring robust regulatory oversight, and addressing the underlying socio-political barriers remain critical steps towards eliminating iodine deficiency disorders across the continent.

### 3.2. Pathophysiological Effects

When the maternal iodine intake is insufficient, a cascade of pathophysiological changes occurs, affecting the function of multiple organ systems, with particularly profound impacts during periods of rapid development such as pregnancy, lactation, infancy, and adolescence (Table 3). Maternal iodine deficiency limits the availability of the substrate needed for the production of T4 and T3, which can lead to the impairment of thyroid hormone synthesis [22]. Reduced thyroid hormone synthesis triggers a compensatory rise in thyroid-stimulating hormone (TSH) via negative feedback mechanisms at the hypothalamic–pituitary axis [40]. Chronic TSH stimulation leads to thyroid hyperplasia and hypertrophy, manifesting clinically as goitre, often the earliest visible sign of iodine deficiency [41].

During pregnancy, the maternal thyroid gland undergoes increased stimulation due to elevated human chorionic gonadotropin (hCG) levels and greater iodine demands [42]. A prolonged insufficient iodine supply during this period exacerbates the risk of maternal hypothyroidism and thyroid enlargement, impairing both maternal and foetal health. Iodine deficiency results in hypothyroidism, characterised by decreased circulating T4 and T3 concentrations [42]. Hypothyroidism leads to a reduction in the basal metabolic rate, causing symptoms such as fatigue, weight gain, cold intolerance, bradycardia, and cognitive slowing [43]. In pregnant women, even subclinical hypothyroidism due to mild iodine deficiency can disrupt pregnancy outcomes, increasing the risk of gestational hypertension, preeclampsia, and placental abruption [42].

Moreover, maternal hypothyroidism negatively affects the intrauterine environment, compromising foetal growth and neurodevelopment [44]. Severe iodine deficiency during pregnancy is associated with increased risks of miscarriage, stillbirth, preterm delivery, and neonatal mortality [34]. The insufficient availability of maternal thyroid hormones critical for foetal growth and organogenesis contributes to these adverse outcomes. Early infant mortality and morbidity are further exacerbated when neonatal hypothyroidism remains undetected in resource-limited settings without systematic newborn screening [34].

The most critical pathophysiological consequence of iodine deficiency arises during foetal and early postnatal brain development [45]. Thyroid hormones are essential for neurogenesis, neuronal migration, synaptogenesis, and myelination. In utero iodine deficiency, especially during the first trimester when the foetus depends entirely on maternal T4, can result in irreversible brain injury, clinically manifesting as cretinism, marked by severe intellectual disability, deaf–mutism, motor spasticity, and growth retardation [46]. Even mild to moderate maternal iodine deficiency has been linked to lower offspring IQ scores, delayed language acquisition, poorer executive function, and suboptimal educational achievement [47]. During lactation, maternal iodine deficiency reduces the iodine concentrations in breast milk, directly compromising the iodine intake of exclusively breastfed infants [48]. Inadequate iodine delivery during infancy, particularly in the first six months, can severely impair early cognitive and psychomotor development that are often irreversible despite the later correction of the iodine status [49].

### 3.3. Cognitive and Developmental Consequences

Despite significant progress in global iodine nutrition, iodine deficiency disorders (IDD) remain widespread, affecting approximately 1.88 billion people, including 241 million school-aged children [50]. This ongoing burden highlights the need for sustained universal salt iodisation programmes and enhanced surveillance, particularly among vulnerable groups such as pregnant and lactating women. For example, during pregnancy, severe iodine deficiency can result in cretinism [51]. Mild to moderate iodine deficiency has been reported to be associated with subtle but significant impairments in offspring’s neurocognitive function, including lower IQ scores, language delays, and poorer educational outcomes [52].

Emerging evidence suggests that in regions with mild to moderate iodine deficiency, both maternal subclinical hypothyroidism and isolated maternal hypothyroxinaemia (characterised by low free T4 concentrations with normal TSH levels) are observed more frequently compared to iodine-sufficient regions [42]. This distinction implies that mild iodine deficiency may selectively impair the maternal thyroid hormone availability without overtly altering the TSH concentrations, thus evading routine thyroid screening. Such subtle disruptions in the maternal thyroid status during early gestation have potential critical implications for foetal neurodevelopment, particularly given the foetus’s complete reliance on the maternal thyroid hormones during the first trimester [53]. Several observational studies have identified associations between maternal hypothyroxinaemia or subclinical hypothyroidism during pregnancy and reduced cognitive outcomes in offspring [34,54,55]. However, these studies have not conclusively established a direct link between thyroid dysfunction and iodine deficiency. It is important to note that thyroid function is a relatively insensitive marker of the population iodine status in adults, complicating the efforts to infer iodine deficiency as an underlying cause of maternal thyroid dysfunction [1].

However, the extent to which mild maternal iodine deficiency during pregnancy influences a child’s neurobehavioural development remains unclear [34]. Several observational studies have consistently shown that mild to moderate maternal iodine deficiency during pregnancy is associated with impaired cognitive outcomes in offspring. Early evidence by Pop et al. demonstrated that maternal hypothyroxinaemia, potentially linked to an inadequate iodine intake, was associated with reduced psychomotor development in infants [56]. Subsequent studies, including those by Velasco et al. and Bath et al., further supported these findings, reporting lower IQ scores, diminished reading comprehension, and poorer psychomotor outcomes in children born to iodine-deficient mothers [5,57]. In the Avon Longitudinal Study of Parents and Children (ALSPAC) cohort study by Bath et al. examining the maternal iodine intake during pregnancy and child neurodevelopment, a low maternal iodine status (i.e., urinary iodine-to-creatinine ratio of <150 µg/g) was associated with an increased risk of suboptimal verbal IQ scores at 8 years of age, as well as poorer reading accuracy, comprehension, and overall reading scores at 9 years. In the study by Velasco et al. [57], children of pregnant women who had received iodine supplements (300 μg/day) from the first trimester onwards (*n* = 133) were compared with children whose mothers had not received iodine supplementation (*n* = 61). Neurodevelopmental assessment was conducted at two years of age using the Bayley Scales of Infant Development. Children born to supplemented mothers had a significantly higher Psychomotor Development Index (PDI) score, with a mean difference of 6.1 points (*p* < 0.02) compared to the non-supplemented group. These associations persisted even after adjusting for multiple potential confounders. Moreover, the findings indicated a dose–response relationship, with progressively poorer cognitive outcomes observed as the maternal iodine status declined. Hynes et al. extended these observations to educational achievement, demonstrating a link between mild maternal iodine deficiency and reduced literacy and numeracy skills at nine years of age [58]. Later, Abel et al. found that suboptimal maternal iodine intake, as estimated from dietary data, was associated with impairments in language, motor, and cognitive development at three years of age in a large Norwegian cohort [59].

Another study in Spain by Murcia et al. reported that in the INMA-Valencia cohort, maternal iodine supplementation ≥ 150 μg/day was associated with a 5.2-point reduction in the Psychomotor Development Index (PDI) (95% CI: −8.1 to −2.2) and 1.8-fold increased odds of low psychomotor development (PDI < 85; 95% CI: 1.0–3.3), compared to an intake < 100 μg/day [60]. Infant neurodevelopment at 1 year was assessed using the Bayley Scales of Infant Development [60]. These associations were stronger in girls and absent in boys. When a wider sample of mother and child pairs from three other regions in Spain were further assessed using the INMA cohort, Rebagliato et al. reported that among both mildly iodine-deficient and iodine-sufficient pregnant women in Spain, prenatal iodine supplementation at doses ≥ 150 μg/day did not result in improvements in infant psychomotor or mental development [61].

A study of 304 children from the Rhea cohort in Greece by Kampouri et al. reported that children with a UIC < 100 μg/L demonstrated significantly lower performance in the motor scale at age 4 (McCarthy Scales of Children’s Abilities (MSCA) motor scale: B = −10.3; 95 % CI: −19.9 to −0.6; *n* = 10) and in non-verbal intelligence at age 6 (Raven’s Coloured Progressive Matrices (RCPM) total score: B = −3.6; 95 % CI: −6.8 to −0.5; *n* = 9) compared with those in the reference group (UIC between 100 and 299 μg/L) [62]. However, no significant associations were observed with the general cognitive scale at age 4, or with the Trail Making Test (TMT) and Finger Tapping Test (FTT) at age 6.

In addition, two other uncontrolled observational studies suggested a potential improvement in psychomotor development, but not in mental development following prenatal iodine supplementation [57,63]. However, the lack of control groups and methodological limitations in these studies constrain the strength of their conclusions. Therefore, these study findings regarding the effects of maternal iodine supplementation in mildly deficient populations are inconsistent.

While some evidence suggests a relationship between the maternal iodine status and early cognitive outcomes, the effect of targeted iodine supplementation during pregnancy in regions of mild deficiency remains uncertain. High-quality, controlled, iodine intervention studies are urgently needed to clarify the role of prenatal iodine supplementation in optimising the neurodevelopment in these settings.

## 4. Maternal Excessive Iodine Intake

### 4.1. Prevalence

In a meta-analysis of eight observational studies with 10,736 pregnant women, Candido et al. reported a substantial variation in the prevalence of excessive iodine intake during pregnancy, which ranged from 3.2% to 98.3% [64]. The pooled estimate indicated that approximately 52% of pregnant women exceeded the recommended iodine intake levels (95% CI: 11–92%), although the analysis demonstrated considerable heterogeneity (*I*^2^ = 99.98%; *p* < 0.001), reflecting marked differences in the study populations, methodologies, and regional iodine exposures [64]. Notably, the highest prevalence of excess iodine intake was reported in a study from East Africa, where 98% of women were found to exceed the intake recommendations (95% CI: 97–99%), highlighting a context of potentially unregulated or high natural iodine exposure [64].

Environmental sources, particularly drinking water, have been identified as significant contributors to excessive iodine intake, especially in endemic regions. Evidence from multiple studies in China demonstrates that pregnant women residing in areas with elevated iodine concentrations in water supplies are at greater risk of iodine excess [65,66,67]. These regions often report iodine concentrations in drinking water exceeding 300 μg/L, substantially above the recommended safety thresholds, and sufficient to induce thyroid dysfunction in vulnerable populations.

Moreover, environmental and geographic characteristics may amplify this risk. Areas classified as having iodine excess are frequently coastal or subject to high rainfall, conditions that may facilitate iodine deposition in soil and water sources through seawater intrusion or flooding [68]. This geochemical enrichment of the water table contributes to chronic exposure in local populations, particularly in rural areas where alternative water sources may be limited. The interplay between hydrogeological conditions and dietary iodine intake thus presents a critical area for public health surveillance and mitigation [64].

### 4.2. Pathophysiological Effects

Although high iodine intakes are generally well-tolerated by most healthy individuals, emerging evidence suggests that a maternal excessive iodine intake may lead to some clinically significant and potentially adverse effects on thyroid function, with downstream implications for foetal and child neurodevelopment [23]. For example, in some susceptible individuals, the use of iodine-containing supplements, fortified foods, or natural sources such as seaweed can lead to excessive iodine intake, which can lead to episodes of maternal hypothyroidism or hyperthyroidism [23]. These effects may be transient in some cases, but in pregnancy, even short-term thyroid hormone disruptions can impair foetal neurodevelopment, especially during the first trimester when the foetus is entirely reliant on maternal T4. This risk is particularly elevated in women with pre-existing thyroid disease, latent autoimmune thyroiditis, or a background of chronic iodine deficiency, where thyroidal autoregulation is compromised.

It is important to note that such risks may extend into the postpartum period as well. During lactation, iodine is actively concentrated in breast milk through the NIS to meet the high requirements of the infant. A maternal excessive iodine intake during this period may lead to elevated iodine concentrations in breast milk, potentially overwhelming the neonate’s immature thyroid gland and increasing the risk of thyroid dysfunction, such as subclinical hypothyroidism. Neonatal thyroid dysfunction during early life has been associated with suboptimal growth and cognitive outcomes, highlighting the need for the vigilant monitoring of the maternal iodine intake throughout both pregnancy and lactation.

#### Thyroidal Adaptation to an Excessive Iodine Intake

During initial exposure, excess iodine enters the thyroid gland via the NIS, triggering the acute Wolff–Chaikoff effect [69]. This effect involves the formation of iodinated inhibitory compounds (such as iodolactones and iodoaldehydes), which transiently inhibit thyroid peroxidase (TPO), an essential enzyme for thyroid hormone production, resulting in reduced thyroid hormone synthesis. In most individuals, the suppression of thyroid hormone synthesis induced by the acute Wolff–Chaikoff effect is transient. In rats, the escape from the acute Wolff–Chaikoff effect is closely associated with a pronounced reduction in the expression of the NIS. Following exposure to excess iodine, a downregulation of NIS expression typically occurs within 24 h, resulting in a marked reduction in intrathyroidal iodine concentrations. This decline in intracellular iodine availability diminishes the formation of iodinated inhibitory compounds. As the concentration of these inhibitory substances decreases, the inhibition on TPO is lifted, allowing for the resumption of normal thyroid hormone synthesis and the restoration of euthyroid status [23].

However, in some susceptible individuals, the failure to escape from the acute Wolff–Chaikoff effect may result in iodine-induced hypothyroidism, which can be either transient or permanent [70]. This impaired adaptation is particularly relevant in individuals with underlying or predisposing thyroid conditions such as Hashimoto’s thyroiditis, Graves’ disease, and postpartum thyroiditis [23]. Of particular concern are the foetus and neonate, whose thyroid glands remain functionally immature for much of gestation and into the neonatal period. This immaturity compromises their ability to escape from the iodine-induced suppression of thyroid function. In utero exposure to excessive maternal iodine, whether through a high dietary intake, supplements, or medical iodine-containing agents, may lead to prolonged foetal or neonatal hypothyroidism, with potentially irreversible consequences for neurodevelopment, especially if unrecognised and untreated in the early postnatal period [23]. However, the underlying mechanisms by which an excessive iodine intake may impair cognitive development remain poorly understood.

### 4.3. Cognitive and Developmental Consequences

An excessive iodine intake, including from iodine supplementation, has been associated with altered thyroid hormone levels during pregnancy in both mothers and neonates [57,71,72,73,74]. Although iodine supplementation during pregnancy is widely recommended to prevent deficiency, evidence regarding its safety at higher intake levels and its long-term effectiveness in supporting optimal child neurodevelopment remains limited. Experimental animal studies have demonstrated that excessive iodine exposure can impair neurodevelopment and brain function in offspring. Rat pups born to dams administered a three-fold higher iodine dose (15–16 μg/day, beginning 12 weeks prior to conception and continued through lactation) exhibited deficits in learning and spatial memory, along with reduced hippocampal expression of brain-derived neurotrophic factor (BDNF), which is a key neurotrophin involved in neuronal survival and differentiation during development [75]. In another experimental study, the offspring of rats exposed to excessive iodine concentrations in drinking water (ranging from 500 to 5000 μg/L) showed decreased brain weight (relative to body weight), elevated oxidative stress in serum, increased hippocampal autophagy, and impaired performance in spatial learning and memory tasks, as assessed by the Morris water maze test. The nature and severity of effects varied across the iodine exposure levels [76]. However, the translational relevance of these findings to humans remains uncertain, as high-quality human data on the potential neurodevelopmental risks associated with iodine excess are notably limited [75].

The few studies published to date have reported inconsistent and sometimes contradictory findings, raising concerns about the potential risks associated with an excessive maternal iodine intake. For example, in the Pregnancy Iodine and Neurodevelopment in Kids (PINK) cohort study of 794 Australian pregnant women, children born to women in both the lowest (<220 μg/day) and highest (>391 μg/day) quartiles of iodine intake during pregnancy exhibited significantly poorer cognitive and language outcomes [77]. Extending these findings within the PINK study cohort, which was conducted in an Australian population that transitioned from mild iodine deficiency to sufficiency following mandatory fortification, a curvilinear relationship was observed between the maternal iodine intake and child neurodevelopment [78]. Specifically, the lowest and highest maternal iodine intakes were associated with the poorest performance in cognitive and language domains. The inflection point for adverse outcomes due to a low iodine intake was approximately 185 μg/day [78]. For excessive intake, cognitive scores declined with intakes exceeding ~370 μg/day, and language scores were adversely affected at intakes above ~350 μg/day [78]. In the Maternal and Infant Nutrition Interventions in Matlab (MINIMat) cohort, an excessive maternal iodine intake during pregnancy as assessed by the UIC (≥500 μg/L) was associated with a tendency towards lower verbal cognitive scores in Bangladeshi children [79]. In contrast, no such association was observed with an elevated iodine status in children at 5 or 10 years of age (UIC ≥ 300 μg/L), suggesting that the prenatal period may represent a particularly sensitive window during which excess iodine adversely affects neurodevelopment [79].

In the IoGeneration cohort from Portugal, children with an elevated iodine status (defined as a urinary iodine-to-creatinine ratio ≥ 250 µg/g) had significantly higher odds of exhibiting below-average IQ scores compared to those with ratios < 250 µg/g, indicating a potential negative association between excessive iodine exposure and cognitive function. Similarly, in the Rhea birth cohort in Greece, Kampouri et al. reported that children with a UIC ≥ 300 μg/L had significantly lower cognitive performance compared to those in the reference group (UIC between 100 and 299 μg/L) [62]. Specifically, these children showed reduced scores on the MSCA at age 4 (B = −3.5; 95 % CI: −6.9 to −0.1; *n* = 101) and on the RCPM at age 6 (B = −1.2; 95 % CI: −2.3 to −0.0; *n* = 98). However, no associations were observed with motor abilities at age 4 or with performance on the TMT and FTT at age 6.

It is important to note that these study findings remain inconsistent across cohorts, and the magnitude of risk appears to vary depending on the iodine source (dietary versus supplemental), timing of exposure during gestation, and baseline iodine sufficiency of the population. Nonetheless, these results underscore the importance of achieving an optimal balance in iodine intake during pregnancy, avoiding both deficiency and excess, to support favourable neurodevelopmental outcomes in children.

## 5. Future Directions and Perspectives

Iodine deficiency remains significantly more prevalent globally than iodine excess. In 2021, only 13 countries were classified as having excessive iodine intakes based on the median UIC of school-aged children, compared with 26 countries identified as iodine deficient and 135 as iodine adequate [4]. While several studies, particularly from European settings, have examined the adverse effects of iodine deficiency on children’s cognitive development, the potential impact of an excessive iodine intake on neurocognitive outcomes has received comparatively limited research attention. Studies examining the effects of excessive iodine on children’s IQ have predominantly been conducted in Asian regions, where naturally high iodine concentrations in drinking water are common [80,81,82]. These studies consistently report lower intelligence scores among children residing in high-iodine areas compared to those from areas with more moderate iodine exposure, suggesting a potential adverse effect of chronic iodine excess on cognitive development.

Although some intervention trials have assessed the impact of maternal iodine supplementation during pregnancy on offspring’s neurocognitive outcomes, the findings from these trials conducted in regions of mild to moderate iodine deficiency have not demonstrated consistent benefits, and uncertainties remain regarding their implications for neurobehavioural development (Table 4). Consequently, the long-term benefits and potential risks associated with maternal iodine supplementation in these populations remain uncertain. While it is well-established that an excessive iodine intake can disrupt thyroid function in susceptible individuals, safe upper intake limits for iodine during pregnancy have yet to be clearly defined. Some studies from high-iodine areas failed to control for non-dietary sources of iodine (water, medications, and disinfectants). It is important to measure and adjust for environmental iodine exposure to isolate dietary effects. There is an urgent need for well-designed, prospective, randomised controlled trials to evaluate the effects of iodine supplementation on both maternal thyroid function and infant neurodevelopment in mildly to moderately iodine-deficient pregnant women. Furthermore, clinical data on the consequences of iodine excess during pregnancy and lactation are critically lacking, limiting the ability to set evidence-based recommendations for a safe chronic iodine intake and supplementation in these vulnerable groups.

Most observational studies investigating the relationship between maternal iodine status during pregnancy and lactation and neurocognitive outcomes in offspring have predominantly used the UIC as a proxy for iodine intake. However, the UIC is recognised as a biomarker suited for assessing the iodine status at the population level rather than at the individual level, due to its considerable intra-individual variability [1]. Consequently, correlating individual UIC measurements with individual neurocognitive test scores is methodologically unsound and risks introducing substantial misclassification bias [87].

The absence of observed benefits from iodine supplementation during pregnancy and lactation may be attributed to several interrelated factors. First, the initiation of iodine supplementation during gestation may be too late to rectify pre-existing iodine deficiency, particularly given the reliance on maternal iodine stores for early foetal thyroid development. Furthermore, the dosage provided by commonly used iodine supplements may be insufficient to restore euthyroid function if maternal stores are already depleted. Second, even a modest but abrupt increase in iodine intake within the recommended levels can induce a transient “stunning” of the thyroid gland, potentially suppressing maternal or foetal thyroid hormone synthesis during a critical developmental window [72]. Severe iodine deficiency is primarily associated with impaired neuromotor development, which represents one of the most consistently affected developmental domains in children [88]. This is of particular concern given that early motor abilities form the foundation for subsequent cognitive, socioemotional, and functional development, including dexterity and skill acquisition later in life [89]. Also, many observational studies did not adequately control for socioeconomic status, dietary habits, or comorbidities, all of which can affect a child’s cognitive outcomes. Future studies should consider implementing multivariate models or propensity score matching to adjust for these confounders.

## 6. Conclusions

The impact of iodine deficiency on child cognitive development has been well-established in the context of severe deficiency; however, growing evidence suggests that even mild to moderate iodine insufficiency during pregnancy and lactation may adversely affect neurodevelopmental outcomes in offspring. Despite this, the existing evidence base remains limited in scope and quality, particularly with regard to the maternal iodine status during these critical periods. High-quality randomised controlled trials (RCTs) and longitudinal cohort studies assessing the impact of maternal iodine nutrition on cognitive development in children remain limited. This gap is especially concerning given that the developing brain is highly sensitive to thyroid hormone levels during gestation and early infancy, and the maternal iodine intake is the primary determinant of thyroid hormone synthesis during pregnancy.

On the other hand, emerging research has begun to highlight the potential harms associated with an excessive iodine intake. A U-shaped relationship has been found between the maternal iodine status and child cognitive outcomes, whereby both deficiency and excess are associated with poorer neurodevelopment. However, data on iodine excess remain limited and are often derived from cross-sectional studies.

Taken together, these findings underscore the importance of achieving and maintaining iodine sufficiency without crossing into excess. There is an urgent need for well-powered, longitudinal cohort studies that explore the full spectrum of iodine exposure, which is from deficiency to excess during pregnancy and lactation with robust neurodevelopmental assessments in offspring. These studies should aim to clarify dose–response relationships and identify safe upper intake thresholds. Also, in order to advance the understanding of iodine’s effects on child neurodevelopment, future research should employ randomised controlled designs, enhanced biomarker panels, standardised outcome assessments, and robust confounder adjustment especially regarding the timing of exposure, thyroid autoimmunity, and sociodemographics.

## Figures and Tables

**Table 1:** WHO classification of iodine status based on median urinary iodine concentration (UIC) in different population groups [3].

Population Group	Median UIC (µg/L)	Iodine Intake Status
Children aged <2 years	<100	Insufficient iodine intake
	≥100	Adequate iodine intake
Children aged 6–12 years	<100	Insufficient iodine intake
	100–199	Adequate iodine intake
	200–299	More than adequate iodine intake
	≥300	Excessive iodine intake
Pregnant women	<150	Insufficient iodine intake
	150–249	Adequate iodine intake
	250–499	More than adequate iodine intake
	≥500	Excessive iodine intake
Lactating women	<100	Insufficient iodine intake
	≥100	Adequate iodine intake

**Table 2:** Daily iodine intakes and tolerable upper intake levels (UL) by life stage [3,23,24,25].

Life Stage	Daily Iodine Intake (µg/Day)			Tolerable Upper Intake Level (UL) (µg/day)	
	US IOM EAR/RDA/AI	UK RNI	WHO/UNICEF/IGN Recommended Intake	US IOM UL	EFSA UL
Infants 0–6 months	110 (AI)	50	90	Not established	Not established
Infants 7–12 months	130 (AI)	60	–	Not established	Not established
Children 1–3 years	65 (EAR)/90 (RDA)	70	–	200	200
Children 4–5 years	–	100	90	–	–
Children 4–8 years	65 (EAR)/90 (RDA)	110	–	300	250
Children 6–12 years	–	130	120	–	–
Children 9–13 years	75 (EAR)/120 (RDA)	140	–	600	450
Adolescents > 12 years	95 (EAR)/150 (RDA)	140	150	900	500
Pregnant women	160 (EAR)/220 (RDA)	140	250	1100	600
Lactating women	200 (EAR)/290 (RDA)	140	250	1100	600

Notes: UK RNI: The Reference Nutrient Intake is the amount of a nutrient that is enough to ensure that the needs of nearly all (97.5%) individuals in a group are being met. AI: Adequate Intake is used when evidence is insufficient to establish a Recommended Dietary Allowance (RDA). EAR: Estimated Average Requirement is the daily intake value estimated to meet the requirement of half the healthy individuals in a specific life stage and gender group. UL: Tolerable Upper Intake Level indicates the maximum daily intake unlikely to cause adverse health effects. ULs for infants are not established due to insufficient data. EFSA’s ULs are generally more conservative compared to those of the US IOM, reflecting regional risk assessments and dietary patterns.

**Table 3:** Consequences of maternal iodine deficiency in pregnancy and lactation [3].

Population Affected	Stage	Consequences
Mother	Pregnancy	Maternal hypothyroidism Goitre Increased risk of miscarriage Increased risk of preterm delivery
Mother	Lactation	Reduced iodine concentration in breast milk Prolonged maternal hypothyroxinaemia Increased risk of postpartum thyroiditis
Foetus	Pregnancy	Impaired neurodevelopment (especially first trimester) Cretinism (severe cases) Intrauterine growth restriction (IUGR) Stillbirth Low birth weight
Infant	Neonatal period	Neonatal hypothyroidism Impaired psychomotor development Increased neonatal morbidity and mortality
Child	Early childhood	Lower IQ scores Language and speech delays Poorer executive function Stunted growth (in severe cases)

**Table 4:** Intervention studies investigating the relationship between maternal iodine status and child cognitive outcomes.

Authors (Year)	Country	Iodine Supplementation (Dosage and Timing)	Infants (*n*)	Child Age at Assessment	Cognitive Tests	Domains Assessed	Main Findings
Velasco et al. (2009) [57]	Spain	300 µg KI vs. control (first trimester of pregnancy)	194	3–18 months	Bayley scales of infant development-I	BDS; PDS; MDS	Higher scores on BDS and PDS (*p* = 0.02) in the iodine-supplemented group.
Santiago et al. (2013) [83]	Spain	Iodised salt in cooking and at the table vs. 200 µg KI vs. 300 µg KI (first trimester of pregnancy)	111	6–18 months	Bayley scales of infant development-III	BDS; PDS; MDS	No significant differences between groups.
Brucker-Davis et al. (2015) [84]	France	Iodine-enriched pregnancy vitamins (150 μg iodine/day) vs. pregnancy vitamins without iodine (first trimester of pregnancy)	44	2 years	Bayley scales of infant development-III	Language, cognitive, and motor	No significant differences between groups.
Zhou et al. (2015) [85]	Australia	150 µg KI vs. placebo (second trimester of pregnancy)	53	18 months	Bayley scales of infant development-III	Language, cognitive, and motor	No significant differences between groups.
Gowachirapant et al. (2009) [86]	India and Thailand	200 µg of potassium iodide (KI) vs. placebo (first trimester of pregnancy)	330	5–6 years	Wechsler preschool and primary scale of intelligence-III	Processing speed, verbal, performance, and full-scale IQ	No significant differences between groups.

Notes: BDS, Behavioural Development Scale; KI, potassium iodide; MDS, Mental Development Scale; PDS, Psychomotor Development Scale.

## Data Availability

No new data were created or analyzed in this study.
